# Human breast milk‐derived exosomes through inhibiting AT II cell apoptosis to prevent bronchopulmonary dysplasia in rat lung

**DOI:** 10.1111/jcmm.17334

**Published:** 2022-07-14

**Authors:** Yahui Zhou, Yiwen Liu, Gen Xu, Lingjie Liu, Huimin Li, Yubai Li, Jing Yin, Xingyun Wang, Zhangbin Yu

**Affiliations:** ^1^ Department of Pediatrics Women’s Hospital of Nanjing Medical University Nanjing Maternity and Child Health Care Hospital Nanjing China; ^2^ Department of Neonatology Wuxi Children’s Hospital affiliated to Nanjing Medical University Wuxi China; ^3^ 117910 The Affiliated Hospital of Xuzhou Medical University Xuzhou Jiangsu China; ^4^ Department of Cardiothoracic surgery First Affiliated Hospital of Nanjing Medical University Nanjing Jiangsu Province China; ^5^ Department of Neonatology Shenzhen People’s Hospital (The Second Clinical MedicalCollege, Jinan University; The First Affiliated Hospital, Southern University of Science and Technology) Shenzhen Guangdong China

**Keywords:** apoptosis, AT II, bronchopulmonary dysplasia, FADD, HBM‐Exo, IL‐17

## Abstract

Human breast milk (HBM) effectively prevents and cures neonatal bronchopulmonary dysplasia (BPD). Exosomes are abundant in breast milk, but the function of HBM‐derived exosomes (HBM‐Exo) in BPD is still unclear. This study was to investigate the role and mechanism of HBM‐Exo in BPD. Overall lung tissue photography and H&E staining showed that HBM‐Exo improved the lung tissue structure collapse, alveolar structure disorder, alveolar septum width, alveolar number reduction and other injuries caused by high oxygen exposure. Immunohistochemical results showed that HBM‐Exo improved the inhibition of cell proliferation and increased apoptosis caused by hyperoxia. qPCR and Western blot results also showed that HBM‐Exo improved the expression of Type II alveolar epithelium (AT II) surface marker SPC. In vivo study, CCK8 and flow cytometry showed that HBM‐Exo improved the proliferation inhibition and apoptosis of AT II cells induced by hyperoxia, qPCR and immunofluorescence also showed that HBM‐Exo improved the down‐regulation of SPC. Further RNA‐Seq results in AT II cells showed that a total of 88 genes were significantly different between the hyperoxia and HBM‐Exo with hyperoxia groups, including 24 up‐regulated genes and 64 down‐regulated genes. KEGG pathway analysis showed the enrichment of IL‐17 signalling pathway was the most significant. Further rescue experiments showed that HBM‐Exo improved AT II cell damage induced by hyperoxia through inhibiting downstream of IL‐17 signalling pathway (FADD), which may be an important mechanism of HBM‐Exo in the prevention and treatment of BPD. This study may provide new approach in the treatment of BPD.

## 1 INTRODUCTION

Bronchopulmonary dysplasia (BPD) is one of the most common complications of premature infants.^1^ Prenatal infection, mechanical ventilation, oxygen poisoning, antioxidant, patent ductus arteriosus (PDA) and postpartum infection can lead to BPD.^2^, ^3^, ^4^ Previous works indicate that systemic corticosteroids improve the respiratory function and accelerate exudation in preterm infants in a short term.^5^ However, newborns treated with glucocorticoid have an increased risk of hypertension, hyperglycaemia and gastrointestinal complications, leading to a long‐term neurodevelopmental delay if glucocorticoid are administered within 4 days of birth.^6^ At present, new approaches are needed to prevent BPD in premature infants.

The survival rate of preterm infants has been increased with the widespread application of oxygen therapy. However, long‐term exposure to hyperoxia levels can interfere with lung development, leading to an irreversible lung dysplasia.^7^ Hyperoxia exposure initially results in destruction of the pulmonary endothelial and epithelial cells, followed by pulmonary oedema, haemorrhage, type II alveolar epithelial cell (AT II) proliferation and fibrosis, ultimately compromising gas exchange.^8^, ^9^

The alveolus is composed of type I (AT I) and type II (AT II) cells. The regeneration of AT I cells has been lost; therefore, the regeneration of AT II cells is critical for the repair of the alveolar structural and functional.^10^, ^11^, ^12^, ^13^ Apoptosis and the inhibition of proliferation of AT II cells can be induced by hyperoxia, which is crucial for the occurrence of BPD.^13^ Thus, the inhibition of AT II apoptosis may help in prevention of BPD occurrence and developing.

A meta‐analysis and retrospective study found that breastfeeding is associated with a lower risk of BPD.^14^, ^15^ Although the effects of human breast milk (HBM) in BPD have been observed, the main physiological mechanism in HBM to prevent BPD is not yet clear.

Exosomes are small endocytic vesicles (30–150 nm) in various body fluids, especially breast milk.^16^, ^17^ In the past few years, the exploration of exosomes has allowed the understanding of the intercellular communication and the therapeutic applications^18^, ^19^ in viral invasion.^16^, ^20^ Exosomes from murine breast milk promote cell growth and proliferation.^21^ HBM‐derived exosomes (HBM‐Exo) protect the necrotizing enterocolitis (NEC).^22^ However, the protective effect of HBM‐Exo on BPD remains unclear.

Our in vivo studies, revealed that HBM‐Exo improved alveolar injury and the expression of AT II cell surface marker surfactant C (SPC) in BPD animal model. Our hypothesis is that HBM‐Exo ameliorates the damage of AT II cells caused by hyperoxia exposure. Therefore, the effect of HBM‐Exo on the damage of AT II cells cause by hyperoxia exposure was evaluated on MLE‐12 cell line caused by hyperoxia. Our results showed that HBM‐Exo exerted a protective effect on AT II cells. RNA‐Seq and rescue experiments revealed that HBM‐Exo inhibited IL‐17 signalling pathway, as well as downstream target FADD, thereby inhibiting cell apoptosis, so as to repair AT‐II cell damage, consequently preventing and treating BPD. Therefore, this study might provide a new approach and ideas in the prevention and treatment of BPD.

## 2 MATERIALS AND METHODS

### 2.1 Collection of samples

The clinical lung tissue samples were obtained from preterm infants who died after medical treatment and family members agreed to autopsy. The HBM samples were collected from the milk bank of Women’s Hospital of Nanjing Medical University. All samples were stored at −80°C until analysed. The collection procedure has been approved by the Women’s Hospital of Nanjing Medical University and the institutional review committee of Nanjing Maternity and Child Care Hospital [Permission No. (2013)78]. The detail information of treating invalid premature infants and mothers who donated HBM was listed in the Tables 1 and 2.

**TABLE 1:** Detail information of mothers who donated human breast milk (HBM)

Sample	Mother	Age	Week	Weight	Delivery way	Parity
	1	30	40 + 1	3430	Natural birth	G2P2
HBM−1	2	27	39 + 1	3180	Natural birth	G1P1
	3	26	38 + 2	3310	Natural birth	G1P1
	4	30	39	3600	Caesarean section	G2P2
HBM−2	5	37	40 + 1	3640	Natural birth	G2P1
	6	30	40 + 3	3590	Natural birth	G2P2
	7	25	41 + 3	3740	Natural birth	G2P1
HBM−3	8	28	41 + 1	3550	Natural birth	G1P1
	9	26	39+3	2800	Natural birth	G1P1

**TABLE 2:** Detail information of treating invalid premature infants

Sample	Week	Weight (g)
Control 1	28 + 1	1120
Control 2	27 + 5	980
Control 3	26 + 2	912
BPD 1	24 + 5	750
BPD 2	26 + 3	821
BPD 3	27 + 4	853

#### 2.1.1 Isolation and identification of human breast milk exosomes

The HBM‐Exo was isolated as previously described.^23^ Briefly, HBMs were collected in sterile test tubes and centrifuged at 3000 g at 4°C for 15 min twice to remove fat layer. Then, the supernatant was transferred to new tubes. The samples were filtered using a 0.22 μM filter. Finally, the filtered liquid was supercentrifuged on a supercentrifuge at 42000 rpm and 4°C for 120 min. After centrifugation, the pellet was clooecte and resuspended in PBS. Nano‐sight analysis (Malvern Instruments) and transmission electron microscopy (TEM) (JEOL 1200EX II) were used to identify HBM‐Exo. Western blot was used to analysed the surface markers of HBM‐Exo, such as CD63 (Proteintech, 1:1000 dilution), CD9 (Proteintech, 1:1000 dilution) and HSP70 (Proteintech, 1:1000 dilution)).

#### 2.1.2 Transmission electron microscopy

HBM‐Exo (3‐5μl) were attached to a formva‐carbon‐coated mesh for 15 s (Electron Microscopy Sciences, Hatfield, PA, USA) for a visual morphological evaluation, then the liquid in excess was removed using a Whatman level 1 filter paper (Sigma‐Aldrich, St. Louis, PA, USA), and they were stained with 2% uranyl acetate for 15 s. Adsorbed HBM‐Exo was detected on JEOL 1010 TEM, and images were recorded with a camera using a 100,000 × magnification (Hamamatsu, Photonics, Hamamatsu City, Japan).^24^

#### 2.1.3 Nanoparticle tracking analysis

Nanoparticle tracking analysis (NS‐300 Nano‐Sight Instrument, Malvern Instruments Ltd., Malvern, UK) were used to measure the Brownian motion of nanoparticles in real time to assess particle size and concentration. The records were recorded using laser microphotography and digital photography systems (sCMOS camera, Hamamatsu Photonics, Hamamatsu, Japan). HBM‐Exo samples were diluted in vesicle‐free PBS. Capture Settings: Camera Level: 5, Shutter: 45, Slider Gain: 15, FPS 25.0 Number of Frames: 1498, Temperature: 22.3°C, Viscosity: (Water) 0.9 cP, Dilution factor: Dilution not recorded. Analysis Settings: Detect Threshold: 5, Blur Size: Auto. Five 30‐s videos at 25 frames per second were recorded, and for each sample, the exosomes were measured three times. Three groups of samples were collected to calculate the distribution, size and average concentration of HBM‐Exo.

### 2.2 Cell culture

Lung AT II cell line (MLE‐12) were purchased from the Chinese Academy of Sciences. The cells cultured in DMEM: F12 medium (Gibco, USA) supplemented with exosome free 10% foetal bovine serum (Gibco, USA), and 1% penicillin and streptomycin (Gibco, USA). Cells were incubated at a 37°C, 5% CO2 human incubator. Control cells were exposed to 21% oxygen (normoxic), while the cells in the vitro hyperoxic model were placed in a special hyperoxic cell culture chamber that exposed to 85% oxygen (hyperoxia). The intervention cells concentration of HBM‐Exo was 15.4 µg/ml protein.

### 2.3 Exosomal labelling (in vitro studies)

Separated HBM‐Exo were labelled with PKH26 accordance to the manufacturer’s protocol (Sigma, USA, No: PHK26GL). The standard dye solution was filtered using a 0.22 μm filter (American microporous, Billerica’s) and again supercentrifuged by supercentrifuge to remove excess dye. MLE‐12 cells were seeded in 6‐well plates for 24 h, then treated with PKH26 marker solution (15.4 µg/ml protein);

### 2.4 Cell proliferation assay

Cell counting kit‐8 (CCK‐8) (Dojindo, Japan) was used to determine cell proliferation according to manufacturer’s instructions. MLE‐12 cells were plated on a 96‐well plate at a concentration of 1×10^3^ cells/well and incubated overnight. The MLE‐12 cells were then treated with HBM‐Exo (15.4 µg/ml protein) at 0, 12, 24, 36 and 48 h. The optical density (OD) value was measured at 450 nm with a microplate reader (BioTek Instruments Inc, Germany).

### 2.5 Cell apoptosis

Annexin V/PI Apoptosis Detection Kit I (BD Biosciences Pharmingen, USA) was used to determine the cell apoptosis. Briefly, 30,000 cells were collected and washed in a 2 mL of cold PBS. Then, 100 mol/L of 4X combined buffer was mixed on the ice. Annexin V 5 ul and Propidium Iodide 5 ul (1 mg/mL) were added, and the cells were incubated on ice for 15 min in the dark. The samples were then diluted with a 4 × 400 μl binding buffer. Cell analysis was performed using flow cytometry (Becton Dickinson, USA) and FlowJo Version 8.6 (Treestar Inc., San Carlos, CA).

#### 2.5.1 Animals

Wildtype SD rats used in this study were purchased from the Animal Center of Nanjing Medical University, and the protocol was approved by the Animal Research and Care Committee of Nanjing Medical University.

#### 2.5.2 Oxygen exposure and rat BPD model

Newborn SD rats with their mothers were kept in a cage in a sealed plexiglass room (China). Exposure to 85% oxygen was performed from postnatal Day 1 (PN1) until PN7 (the alveolar phase corresponding to lung development in newborn SD rats) and a period of recovery was allowed. In this experimental model, the lungs of SD rats at PN7 had a pathological phenotype minicking severe BPD in humans.

#### 2.5.3 Preparation of human breast milk exosomes for injections

A supplementary HBM‐Exo (200 μg/mL protein) formulas were prepared and the experimental group was treated by a intragastric administration.^16^, ^17^, ^18^ Hyperoxia and control groups remained with their mothers were breastfeeding. On the PN7, lung tissues were collected, and some of them fixed in 4% paraformaldehyde solution, embedded in paraffin, cut into 5‐μm sections and stained with haematoxylin and eosin (H&E) for microscopic evaluation.

### 2.6 In vivo biodistribution of human breast milk‐ exosomes (HBM‐Exo)

HBM‐Exos were administered by gavage as described above. Briefly, HBM‐Exos were labelled with fluorescent dye DiR (1 μM). SD rats 7 days old were treated with Dir labelled HBM‐Exos (200 μg /mL protein by intragastric gavage (i.g.). The rats gavaged with salinewere used as controls. In Vivo imaging system (IVIS Spectrum, PerkinElmer, USA) was used to assess the fluorescence intensity of the whole rats, and different organs were collected to image them.

### 2.7 Western blotting

The Sample size of Western blot in Figure 2 was *n* = 6; the sample size for Western blot in Figure 6 was *n* = 3. The cell treated and untreated with HBM‐Exo were lysed on ice for 30 min using the radioimmunoprecipitation test (RIPA) buffer containing protease inhibitor cocktail and phosphatase inhibitor (Transgen Biotech, Beijing, China), The samples were centrifuged at 12000 rpm and 4°C for 30 min. The BCA kit was used to measure the protein concentration, 20–40 µg of the lysate for gel electrophoresis, and the proteins were transferred to a polyvinylidene fluoride membrane (Millipore, Billerica, MA, USA). The membrane was treated with 5% skim milk and incubated at room temperature for 2 h. The primary antibodies were diluted according to the manufacturer’s instructions and the membrane was treated with the following antibodies and incubated at 4°C overnight:: SPC (Proteintech, 1:1000 dilution), β‐actin (Proteintech, 1:1000 dilution), CD63 (Proteintech, 1:1000 dilution), CD9 (Proteintech, 1:1000 dilution), HSP70 (Proteintech, 1:1000 dilution), IL‐17 (Proteintech, 1:1000 dilution), FADD (Proteintech, 1:1000 dilution), caspase3 (Proteintech, 1:1000 dilution) and caspase9 (Proteintech, 1:1000 dilution). The membrane was washed three times with TBS‐T, subsequently treated with secondary antibodies (goat anti‐rabbit or goat anti‐mouse isotype: IgG (Proteintech 1:5000) HRP‐conjugate); and incubated at room temperature for 1 h. An enhanced chemiluminescence detection kit was employed to develop blots and ChemiDocTM XRS + Imager‐Bio‐Rad (Hercules, CA, USA) was used to capture the images.

### 2.8 Immunofluorescence

The cells were fixed with 4% paraformaldehyde, infiltrated with 0.2% Triton X‐100/PBS and sealed with 5% BSA/PBS. Cells were then treated with SPC antibody (Proteintech, 1:200 dilution)diluted with 1% BSA, and incubated overnight in a refrigerator at 4°C. The next day, the cells were washed with PBS 3 times at 4°C, 5 min each time, and then incubated with a fluorescent secondary antibody (Goat Anti Rabbit, 1:1000) for 2 h. Next, they were washed with PBS for 3 times, stained with DAPI (1:1000, 5 min), washed with PBS for 3 times, and finally photographs were taken using a fluorescence microscope (Zeiss, Germany).

### 2.9 Lung preparation, histology, and immunohistochemistry

The rats were sacrificed on Day 7 and lungs were collected. The right lung was fixed with 4% paraformaldehyde (pH 7.4, 20 cm H_2_O) for at least 2 h, divided into 3 parts from the top to the bottom of the lung, cut into 3 μm‐thick and sections and stained with H&E. As regards Immunohistochemistry (IHC) 4‐μm‐thick sections were cut and dewaxed. The antigen was extracted in 10 mM citric acid buffer at pH 6.0 and the cestion was placed in a pressure cooker for 10 min. Endogenous peroxidase activity was inhibited using 0.5% H_2_O_2_/methanol solution for 15 min, and then blocked with 1.5% rabbit serum in PBS (Science cell, China) for 30 min. Slides were then treated with the primary antibody goat anti‐mouse Ki67 (Proteintech, 1:100 dilution) and caspase3 (Proteintech, 1:100 dilution) and incubated at room temperature for 1 h. Next, they were incubated with the secondary antibody, followed by diaminobenzidine staining according to the instruction of the Vectastain kit. Sections were counterstained with Harris haematoxylin and scanned in an Aperio scanner. (ePathology Solutions).

### 2.10 RNA isolation and quantitative real‐time PCR

Total RNAs were extracted with the TRIZOL (Life, USA) and RNAeasy kit (Tiangen, China). The first strand of cDNA was synthesized by iScript cDNA Synthesis kit (Takara, Japan) according to the instructions for qPCR. The mixed cDNA samples were used for PCR reaction with SYBR Green (Life, USA) in 20 mol/L volume. The primer used are listed s in Table 3. The reaction was performed at 95°C for 10min, followed by denaturation at 94°C for 15 s, annealing at 54°C for 30 s and extension at 72°C for 40 s, with a Real‐Time System (Applied Biosystems, USA).

**TABLE 3:** Primer sequences of circRNAs

Primer name	Sequence
Rat SPC‐F	GAGATGAGCATCGGAGGAGC
Rat SPC‐R	AGGAGCCGCTGGTAGTCATA
Rat β‐actin‐F	CAGGGTGTGATGGTGGGTATGG
Rat β‐actin‐R	AGTTGGTGACAATGCCGTGTTC
Mouse SPC‐F	ATGGAGAGTCCACCGGATTAC
Mouse SPC‐R	ACCACGATGAGAAGGCGTTTG
Mouse β‐actin‐F	CCACAGCTGAGAGGGAAATC
Mouse β‐actin ‐R	TCTCCAGGGAGGAAGAGGAT

### 2.11 RNA‐Seq and bioinformatics analysis

Total RNAs were extracted from MLE‐12 cells using a RNeasy kit (Qiagen, China). The library construction was performed according to the the Shanghai Bohao Institute’s standard procedure. Subsequently, Illumina HiSeq 2000 platform was used for high‐throughput sequencing. The resulting reads were aligned to those the RefSeq database (ftp://ftp.ncbi.nih.gov/refseq) using Bowtie2 (Langmead and Trapnell et al., 2009) after removing sequences with low quality, unknown bases (more than 10%) and adapters and mapped to the reference genome using BWA (Li and Durbin, 2009). The differentially expressed genes (DEGs) were identified as NOISeq according to the following criteria: fold change≥1.5 and *p* < 0.05. The gene ontology (GO) enrichment analysis (http://www.geneontology.org/) was performed to find all GO terms that were enriched in DEGs, and KEGG (http://www.genome.jp/kegg/) was used to analyse in which DEGs were enriched (Kanehisa and Araki et al., 2008).

### 2.12 Statistical analysis

Data were expressed as mean ±standard deviation (SD). The statistical significance between two groups were achieved by using two‐tailed student’s *t*‐*test*, where appropriate, while multiple comparisons were performed using one‐way ANOVA. *p* < 0.05 was considered statistic significant.

## 3 RESULTS

### 3.1 Characterization of HBM‐Exo

**FIGURE 1:**
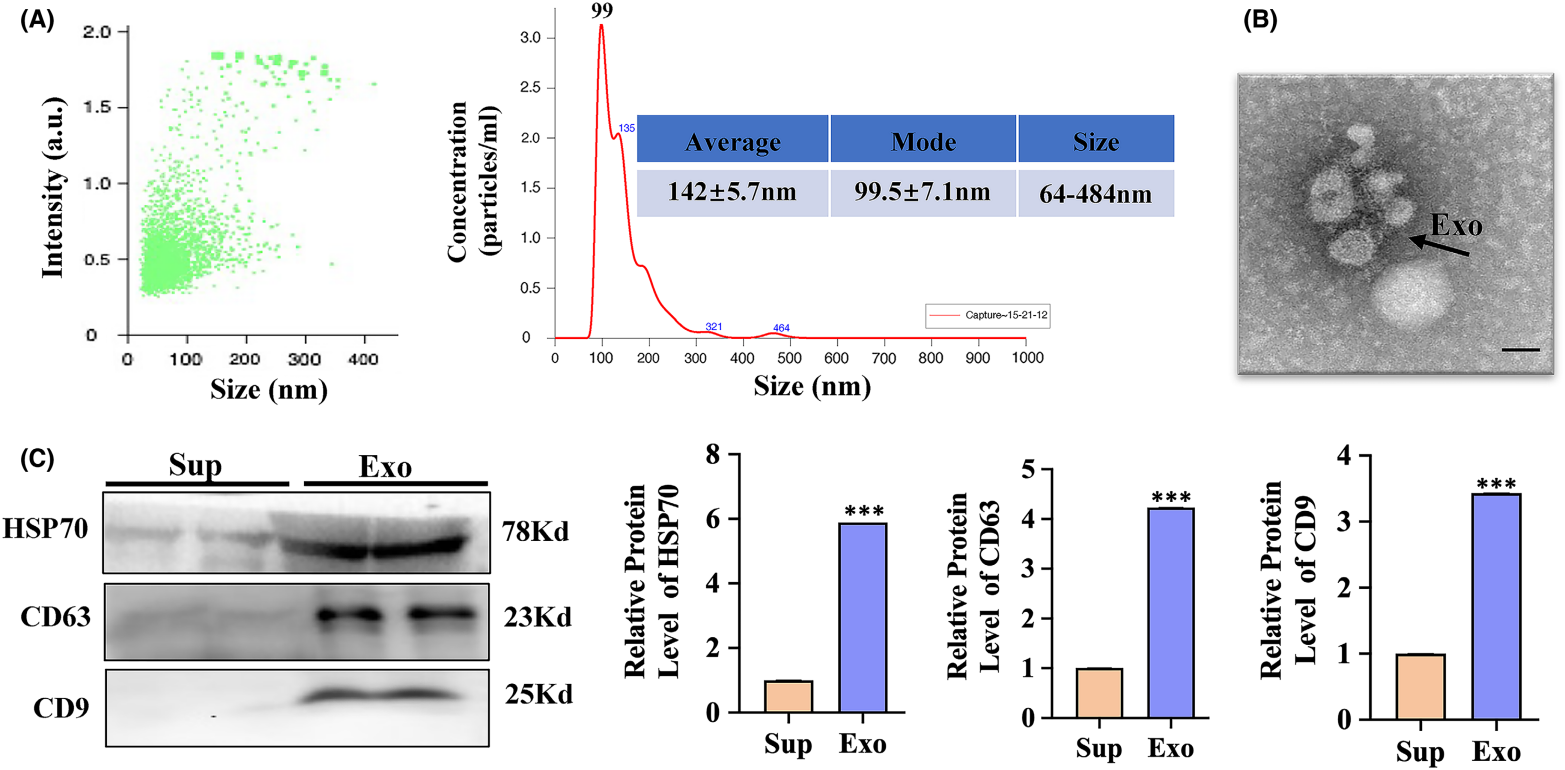
Characterization of human breast milk (HBM)‐Exo. (A) Nano‐Sight analysis was used to determine the average size and strength of exosomes. (B) Representative microscope images of HBM‐Exos. The sample was fixed and analysed using electron microscopy. The scale is 100 nm; (C) expression of CD63, HSP70 and CD9 markers in HBM‐Exos by Western blot. Unpaired t‐test, *p < 0.05, **p < 0.01, ***p < 0.001. Exo: HBM‐Exo, Sup: Supernatant. *VS Sup

The average diameter of purified HBM‐Exo was 30‐150nm by Nano‐sight tracking and electron microscopy analysis, (Figure 1A,B), which was consistent with the previously known characteristics of HBM‐Exo. HBM‐Exo membranes were rich in endosome‐specific markers, such as CD9, CD63 and HSP70 (Figure 1C). These results suggested that the isolation of exosome from HBM was successful.

### 3.2 Effects of HBM‐Exo in animal model

**FIGURE 2:**
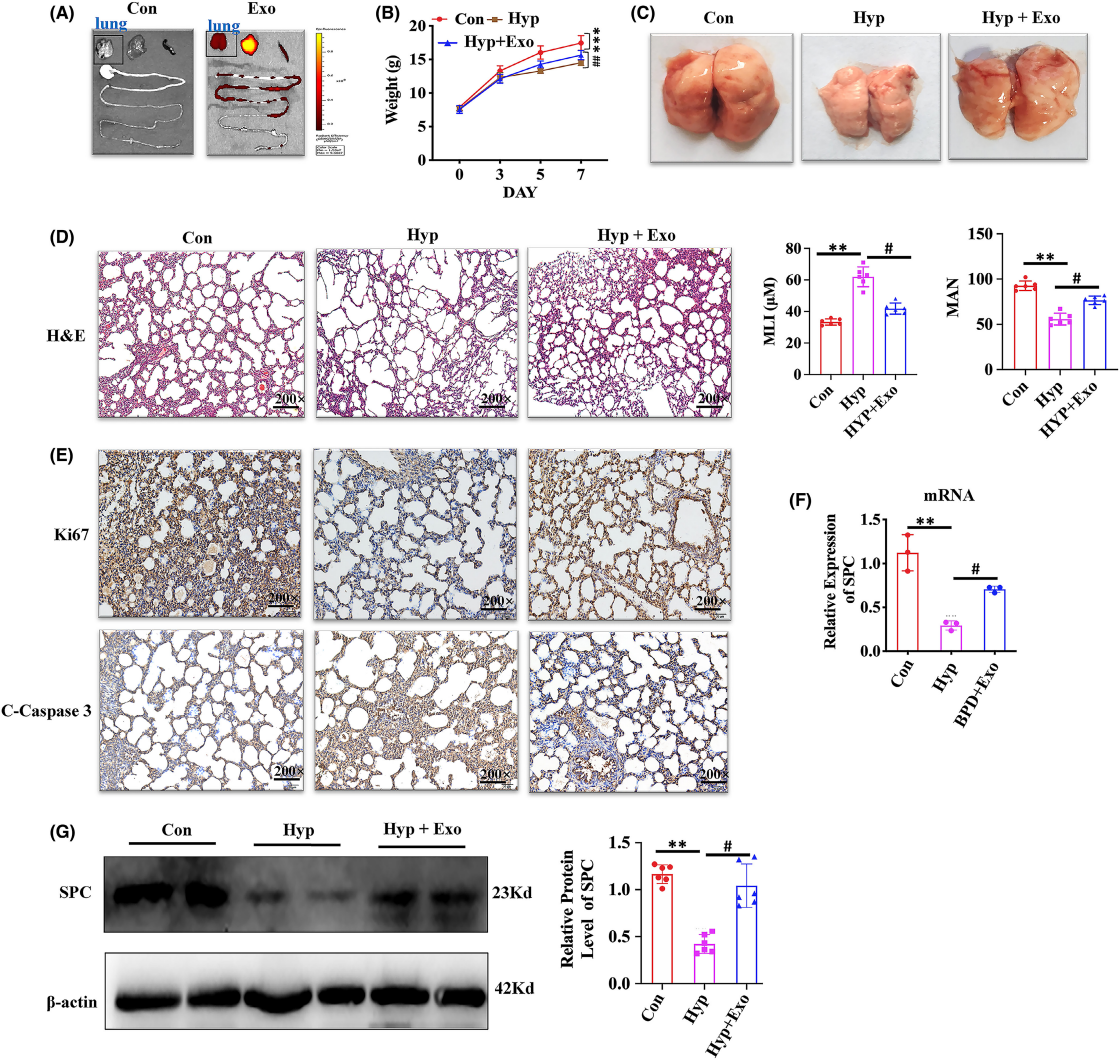
Human breast milk (HBM)‐Exo Function in Animal Model. (A) Fluorescence imaging of a mouse model at 12 h post‐administration of DiR‐labelled HBM‐Exo. (B) The body weight of control, hyperoxia and hyperoxia with HBM‐Exo treatment group. (C) The morphology of lung tissue in the control, hyperoxia and hyperoxia with HBM‐Exo treatment group. (D) Histopathological changes in the lung by H&E, quantification of mean linear intercept (MLI) represents a surrogate of average air space diameter, quantification of mean alveolar number (MAN) represents the average number of alveoli, data represent results from 6 individual studies. Micrographs are representative and were obtained at the same magnification. (E) Representative images of Ki67 immunostaining showing the proliferation and the C‐caspase3 immunostaining showing the apoptosis (brown staining) in the control, hyperoxia and hyperoxia with HBM‐Exo treatment group. Micrographs are representative and were obtained at the same magnification. (F) SPC mRNA relative expression in the control, hyperoxia and hyperoxia with HBM‐Exo treatment group, data represent results from 3 individual studies. (G) SPC protein relative expression in the control, hyperoxia and hyperoxia with HBM‐Exo treatment group, data represent results from 6 individual studies. The pictures shown are representative. Unpaired t‐test, */#*p* < 0.05,**/##*p* < 0.01, ***/###*p* < 0.001. Con: control, Exo: HBM‐Exo, Hyp: Hyperoxia, C‐Caspas: cleaved‐caspase. * VS Con, # VS Hyp. The concentration of HBM‐Exo: 200 μg/ml protein

We found that Dir‐labelled HBM‐Exo target lung tissue in vivo at 12 h after administration (Figure 2A); The weight of newborn rats in the BPD group increased less than that of rats in the control group after 7 days of high oxygen exposure, but the weight of newborn rats treated with HBM‐Exo during high oxygen exposure improved significantly (Figure 2B). Pictures of the newborn rat lung tissue under high oxygen (85%) exposure and stained with H&E were taken at Day 7. The morphology of the lung tissue revealed that the lung tissue structure in the hyperoxia exposure group collapsed significantly than in the control group, and HBM‐Exo treatment significantly improved the collapse caused by hyperoxia exposure (Figure 2C). H&E staining showed that the alveolar septa was enlarged and the number of alveoli was decreased due to hyperoxygen exposure, effect that was also alleviated in the HBM‐Exo intervention group (Figure 2D). The IHC results showed that HBM‐Exo treatment also improved the inhibition of Ki67 and the increase of cleaved‐caspase 3 (C‐Caspase3) induced by high oxygen exposure (Figure 2E).; The qPCR and Western blot detection the SPC expression of AT II markers was significantly decreased, but the damage was significantly ameliorated by the treatment with HBM‐Exo (Figure 2F,G). This results indicated that HBM‐Exo might prevent and cure BPD by repairing AT II cells damage caused by high oxygen exposure.

### 3.3 Effects of HBM‐Exo in AT II cells

**FIGURE 3:**
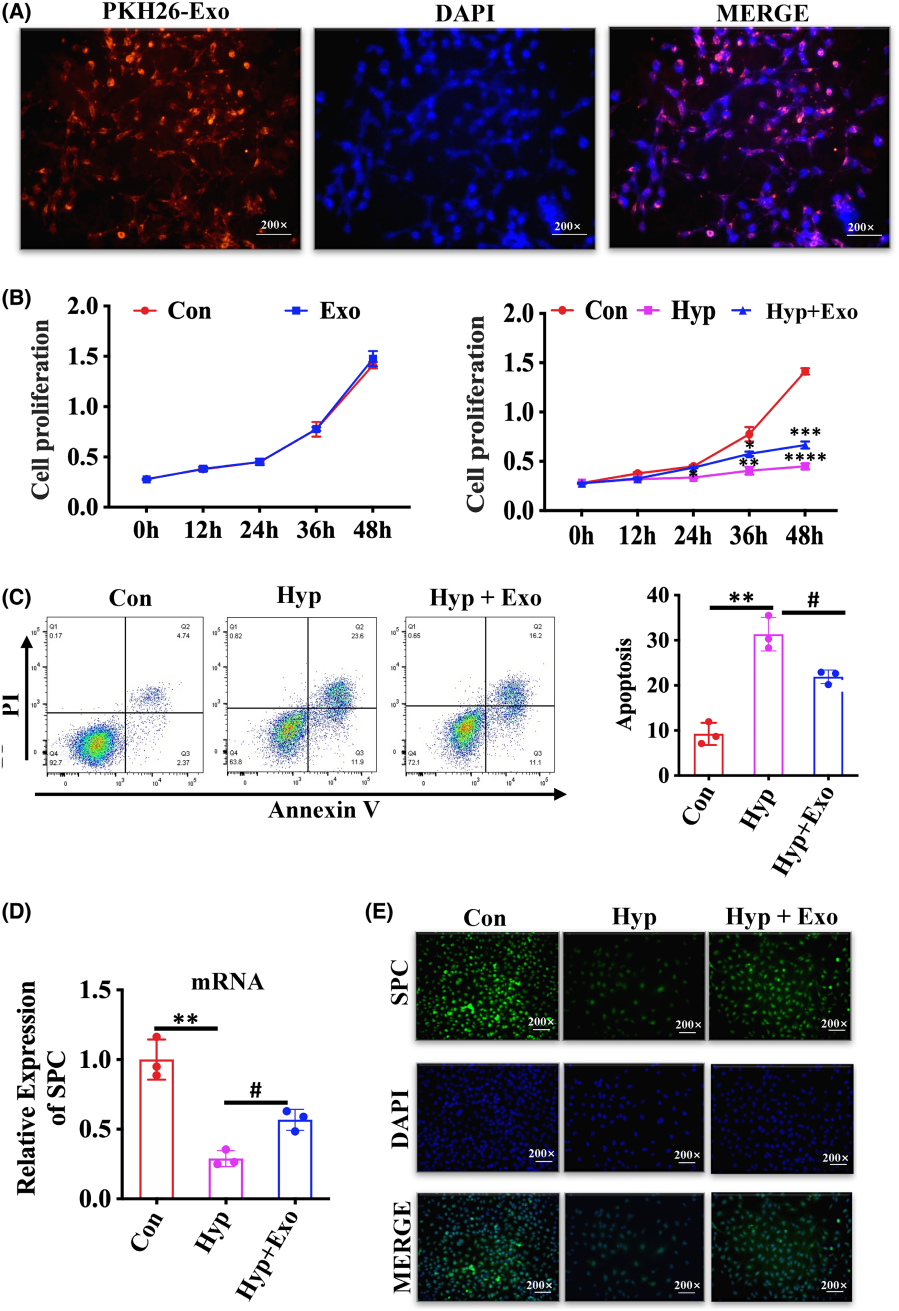
Human breast milk (HBM)‐Exo Function in the AT II cells. (A) Cellular internalization of HBM‐Exo in MLE‐12 cells. The PKH26‐labelled HBM‐Exo were incubated with MLE‐12 cells for 6 h (red) and stained with DAPI (blue). Original magnification: 200x. (B) Proliferation curves of MLE‐12 cells co‐cultured with HBM‐Exos, data represent results from 3 individual studies. (C) The apoptosis rates of MLE‐12 cells following co‐culture with HBM‐Exo, data represent results from 3 individual studies. (D) qPCR evaluate the relative expression of SPC mRNA, data represent results from 3 individual studies. (E) Immunofluorescence evaluate the expression of SPC protein. Original magnification: 200x. The pictures shown are representative. Unpaired t‐test, */#*p* < 0.05,**/##*p* < 0.01, ***/###*p* < 0.001. Con: control, Exo: HBM‐Exo, Hyp: Hyperoxia, * VS Con, # VS Hyp. The concentration of HBM‐Exo: 15.4 μg/ml protein

The AT‐II cell line MLE‐12 was selected to for further verify the effect of HBM‐Exo on the repair of AT‐II cell damage. First, MLE‐12 cells were co‐incubated with pKH26‐labelled HBM‐Exo, and the result showed that the labelled HBM‐Exo were easily internalized, as shown in the image taken at 6 h (Figure 3A). CCK8 results showed that the HBM‐Exo significantly reduced the inhibition of MLE‐12 cell proliferation caused by hyperoxia exposure. In addition, no cytotoxic effect was exerted by the HBM‐Exo, and the improvement effect on hyperoxia‐induced MLE‐12 cell damage was time‐dependent (Figure 3B). Furthermore, flow cytometry results showed that the HBM‐Exo significantly reduced hyperoxia‐induced apoptosis (Figure 3C). Further study revealed that the expression of SPC, a marker on the surface of AT II cells, which was reduced by hyperoxia, was increased by HBM‐Exo. (Figure 3D,E). These results suggest that HBM‐Exo repaired AT II cell damage caused by high oxygen exposure.

### 3.4 General properties of the differentially expressed genes

**FIGURE 4:**
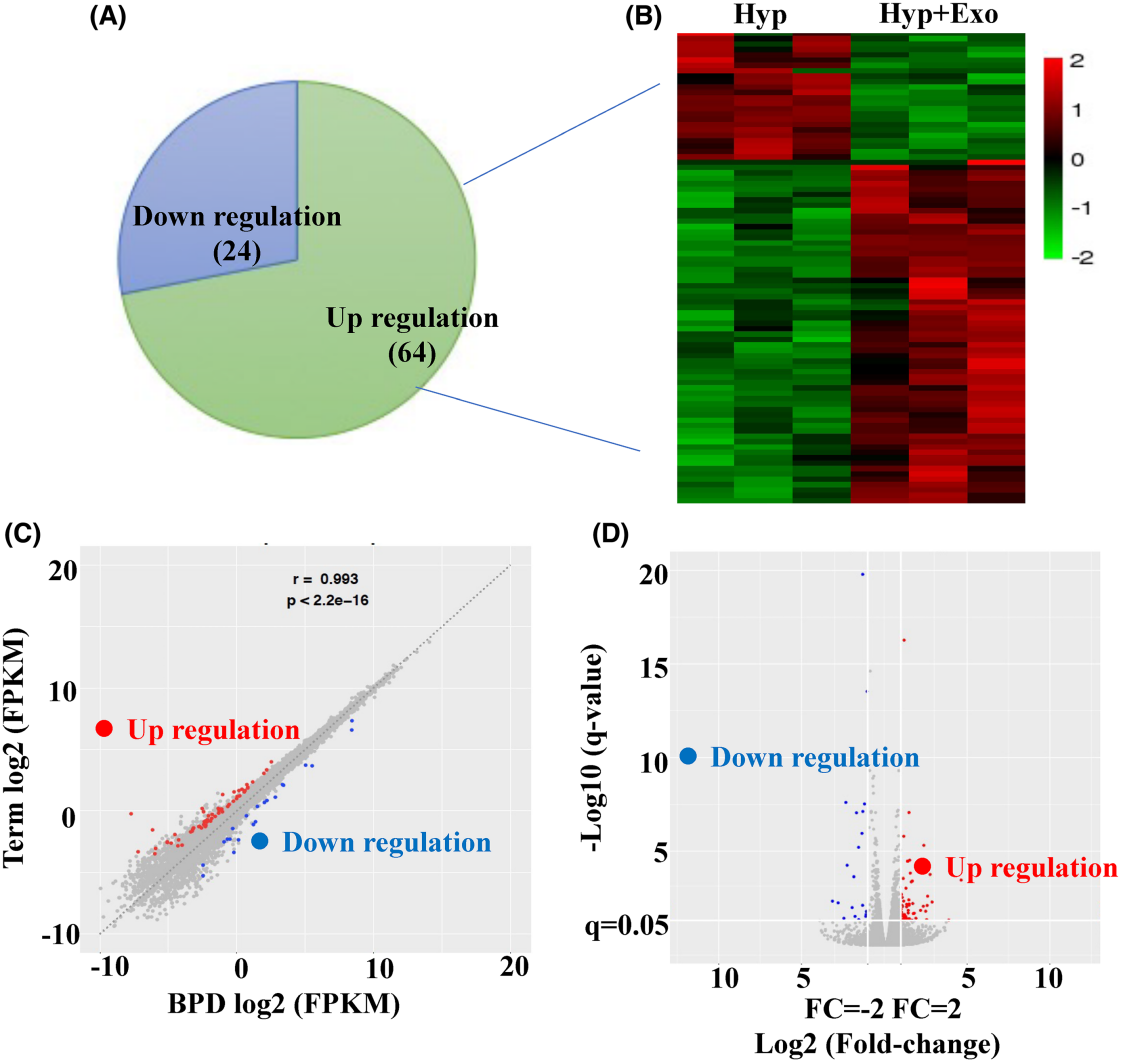
General Properties of the differentially expressed genes. (A) The number of DEGs. (B) Heatmap analysis of the genes. (C) Scatter plot showing the significantly changed genes identified in two groups. (D) Volcano plot showing the significantly changed genes identified in two groups. (Red dots indicate significantly up‐regulated genes; blue dots indicate significantly down‐regulated genes)

The differences in mRNA expression between the hyperoxia exposure group and the HBM‐Exo+hyperoxia exposure group were evaluated in MLE‐12 cells to further confirm the potential molecular mechanism of HBM‐Exo in the prevention and treatment of AT II cell damage by high oxygen exposure. The results revealed that a total of 88 mRNAs were significantly different:24 were up‐regulated and 64 were down‐regulated (Figure 4A,B) (Supplementary Table 1). The detected mRNAs were also shown in the scatter plots and volcanic maps (Figure 4C,D).

### 3.5 GO and KEGG pathway analysis

**FIGURE 5:**
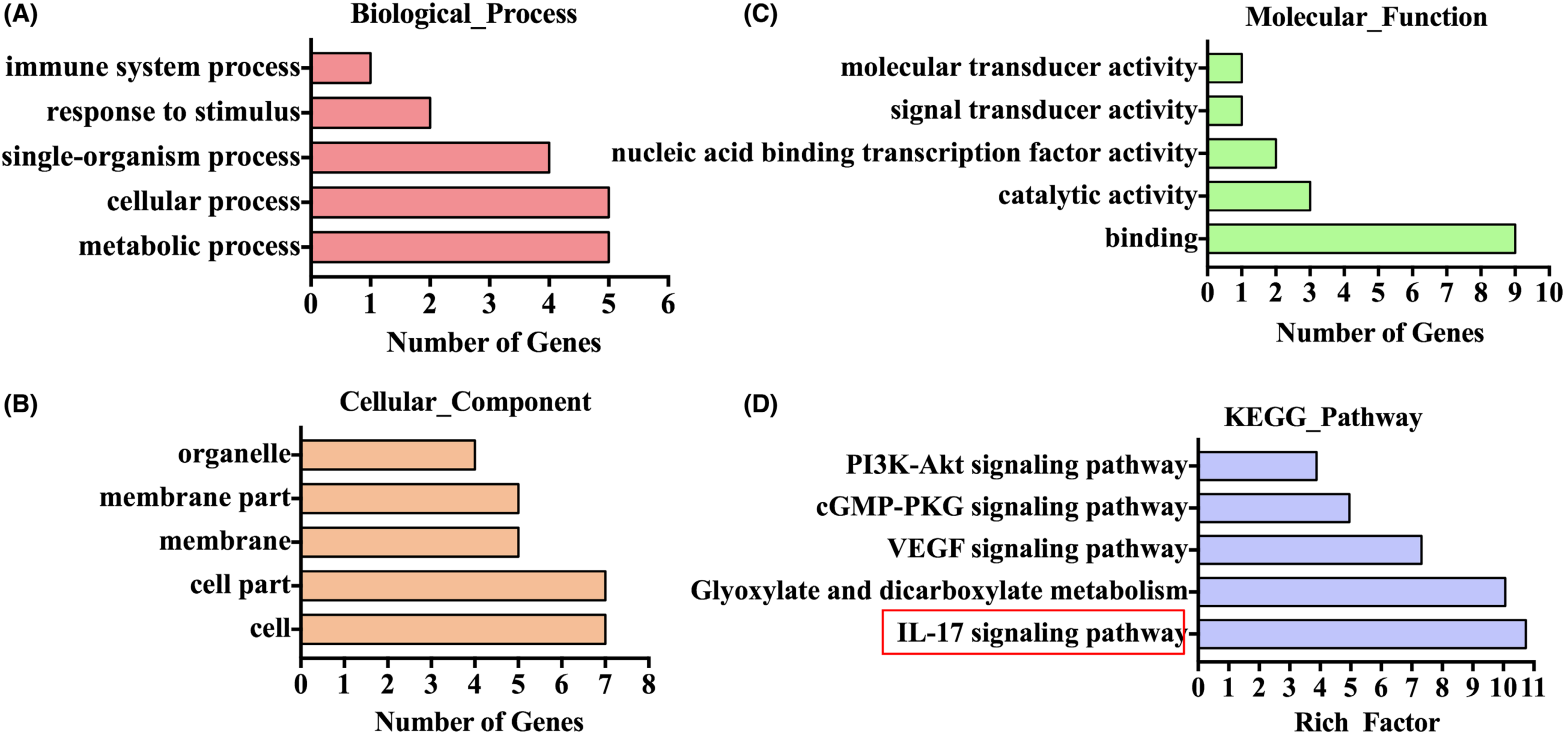
GO and KEGG Pathways Analysis. (A) Biological process. (B) Cellular component (C) Molecular function (D) KEGG Pathway analysis

GO and KEGG pathway analysis was carried out to annotate potential functions of the HBM‐Exo. The biological process analysis showed that HBM‐Exo were involved in immune system process, response to stimulus, single‐organism process, cellular process and molecular process (Figure 5A). Cellular component analysis showed that these functions were significantly enriched in organelle, membrane pat, membrane, cell part and cell. In terms of molecular function (Figure 5B), the most significantly enriched GO term was molecular transducer activity, signal transducer, nucleic acid binding transcription factor activity, catalytic activity and binding (Figure 5C). The main signalling pathways was IL‐17 signalling pathway (Figure 5D).

### 3.6 HBM‐Exo decreased the FADD and apoptosis expression

**FIGURE 6:**
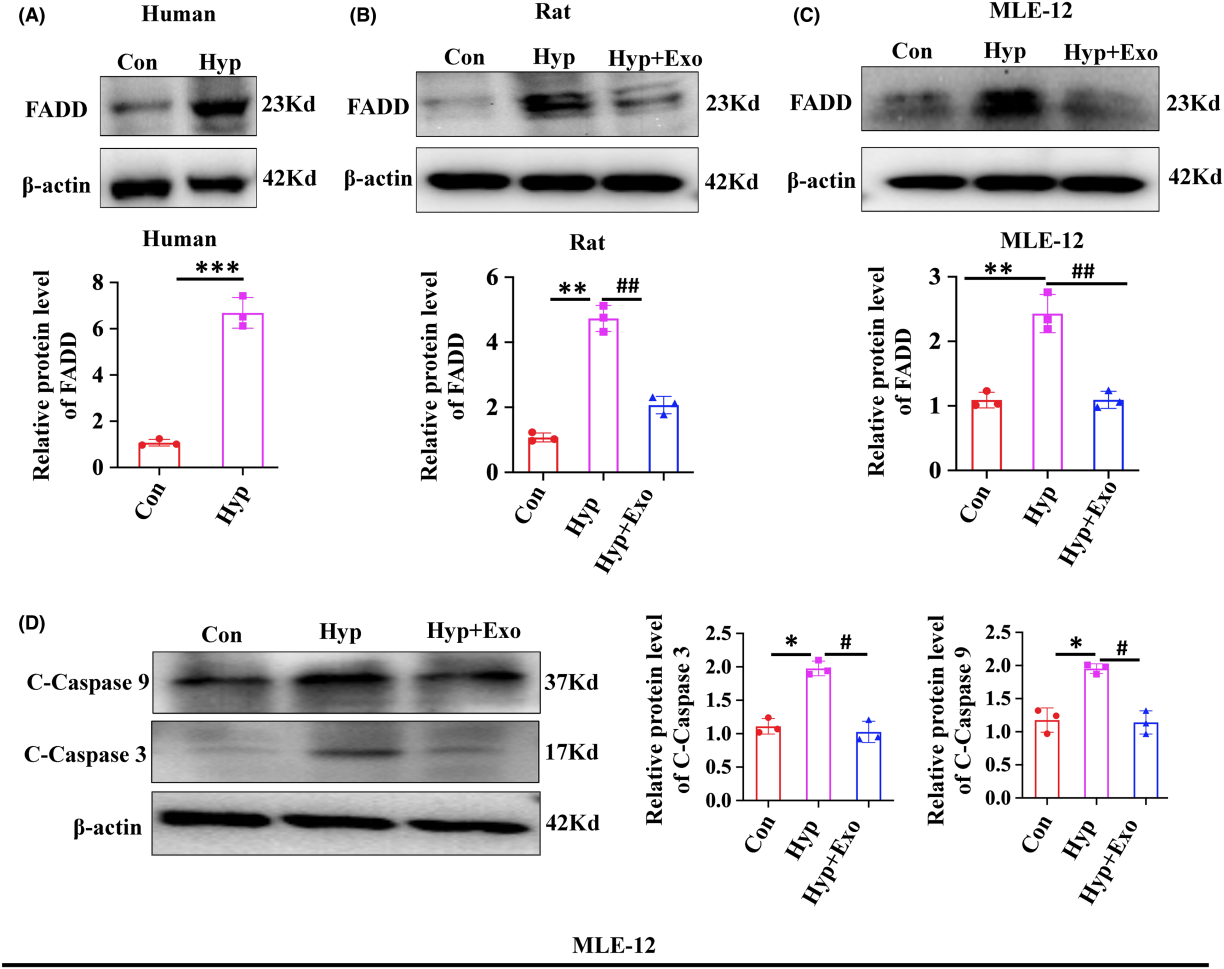
Human breast milk (HBM)‐Exo Decreased the FADD and Apoptosis Expression. (A) FADD protein expression in clinical lung tissue samples by Western blot; (B) FADD protein expression in rats by Western blot; (C) FADD protein expression in cells by Western blot; (D) Protein expression of apoptosis‐related C‐Caspase3 and C‐Caspase9 in cells by Western blot. Data represent results from 3 individual studies. The pictures shown are representative. Unpaired t‐test, */#*p* < 0.05,**/##*p* <.01, ***/###*p* < 0.001. Con: control, Exo: HBM‐Exo, Hyp: Hyperoxia, C‐Caspas: cleaved‐caspase. * VS Con, # VS Hyp. The concentration of HBM‐Exo: 15.4μg/ml protein

GO analysis showed that the enrichment of IL‐17 signalling pathway was the most significant. Thus, our hypothesis was that HBM‐Exo might inhibit hyperoxia‐induced AT‐II cells apoptosis by this pathway. FADD is a downstream target of the IL‐17 signalling pathway and its expression was increased in clinical BPD lung tissue samples (Figure 6A); The expression of FADD was also increased in animal and cell that exposed to hyperoxia and was decreased by HBM‐Exo treatment (Figure 6B,C); Western blot also showed that the C‐Caspase 3 and C‐Caspase 9 was significantly up‐regulated after hyperoxia exposure, while they were significantly down‐regulated after HBM‐Exo treatment (Figure 6D).

### 3.7 Activation of IL‐17 signalling pathway inhibits the mechanism improvement of HBM‐Exo

**FIGURE 7:**
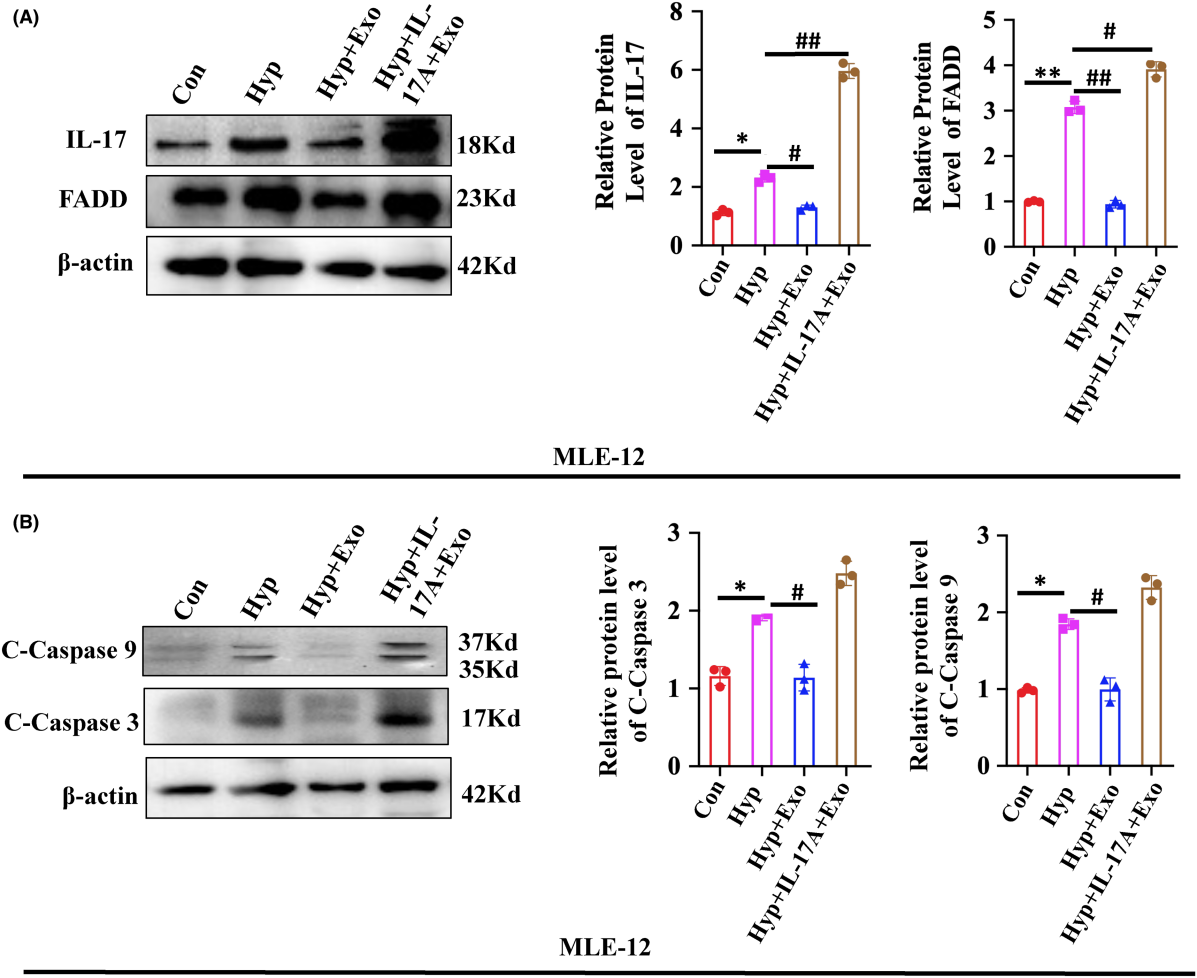
Activation of IL‐17 signalling pathway inhibits the mechanism improvement of human breast milk (HBM)‐Exo. (A) IL‐17 and FADD protein expression in control, hyperoxia and hyperoxia with HBM‐Exo treatment group, as well as hyperoxia with HBM‐Exo treatment plus IL‐17 cytokines group by Western blot. (B) Protein expression of the apoptotic‐related C‐Caspase3 and C‐Caspase9 in control, hyperoxia, hyperoxia with HBM‐Exo treatment group, as well as hyperoxia with HBM‐Exo treatment plus IL‐17 cytokines group by western blot. Data represent results from 3 individual studies. The pictures shown are representative. Unpaired t‐test, */#*p* < 0.05,**/##*p* < 0.01, ***/###*p* < 0.001. Con: control, Exo: HBM‐Exo, Hyp: Hyperoxia, C‐Caspas: cleaved‐caspase. * VS Con, # VS BPD. The concentration of HBM‐Exo: 15.4μg/ml protein

We further activated the IL‐17 signalling pathway by adding IL‐17A cytokines to MLE‐12 cells. The results showed that the promoting effect of HBM‐Exo on FADD disappeared after the activation of the IL‐17 signalling pathway. (Figure 7A). Moreover, the inhibitory effect of HBM‐Exo on the C‐Caspase 3 and C‐Caspase 9 also disappeared due to the activation of the IL‐17 signalling pathway (Figure 7B). All the above results indicate that the HBM‐Exo down‐regulated cell apoptosis by inhibiting the IL‐17 signalling pathway, thus improving the induced AT II cell injury.

### 3.8 Activation of IL‐17 signalling pathway inhibited the effect of HBM‐Exo in ATII cells

**FIGURE 8:**
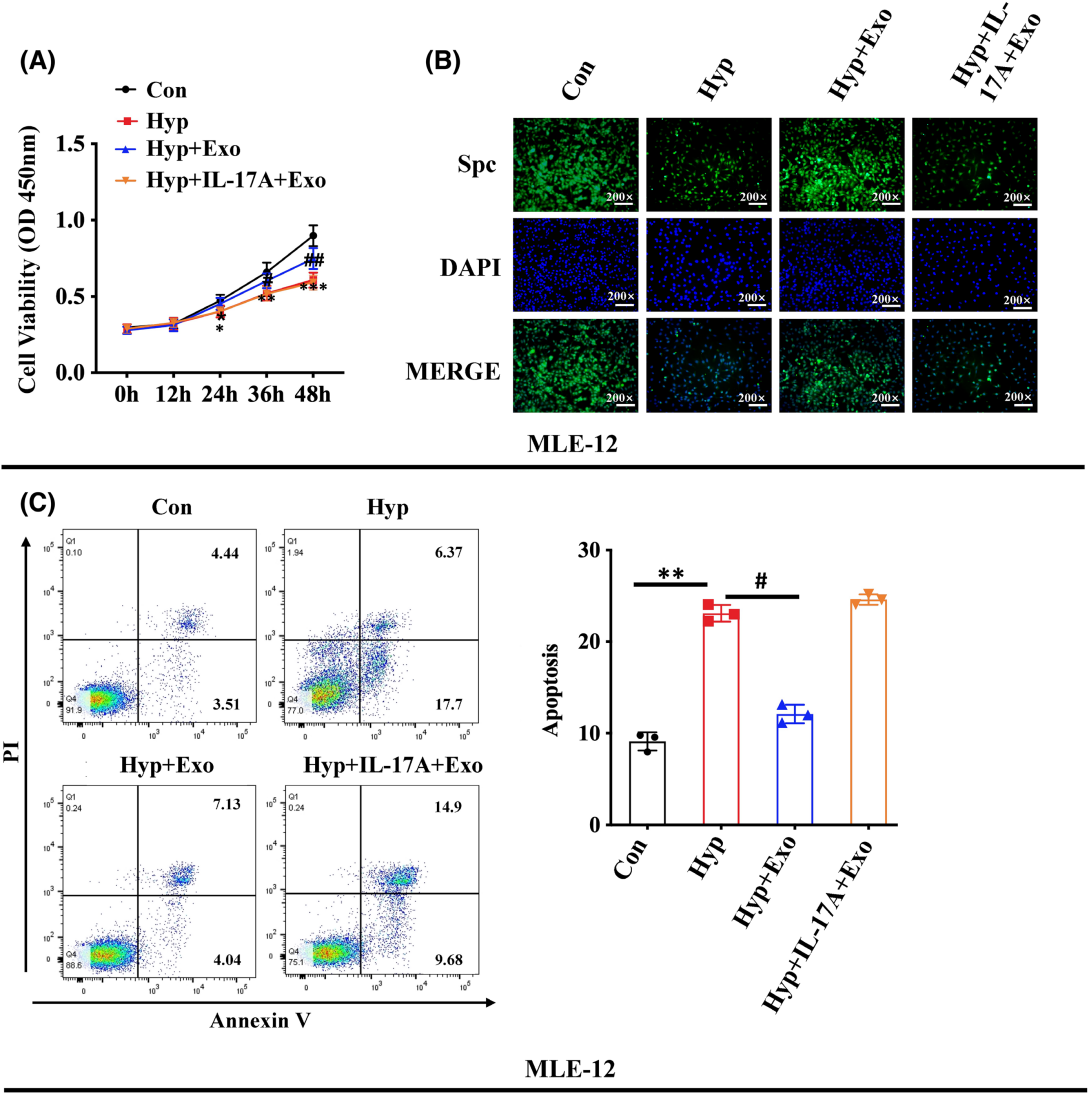
Activation of IL‐17 signalling pathway inhibits the function improvement of human breast milk (HBM)‐Exo. (A) Cell proliferation in the control, hyperoxia, hyperoxia with HBM‐Exo treatment group, as well as hyperoxiawith HBM‐Exo treatment plus IL‐17 cytokines group by CCK8 kit; (B) SPC protein expression in the control, hyperoxia and hyperoxia +HBM‐Exo treatment group, as well as hyperoxia with HBM‐Exo treatment plus IL‐17 cytokines group by immunoflurescenc. (C) Cell apoptosis in the control, hyperoxia, and hyperoxia with HBM‐Exo treatment group, as well as hyperoxia with HBM‐Exo treatment plus IL‐17 cytokines group by flow cytometry. Data represent results from 3 individual studies. The pictures shown are representative. Unpaired t‐test, */#*p* < 0.05, **/##*p* < 0.01, ***/###*p* < 0.001. Con: control, Exo: HBM‐Exo, Hyp: Hyperoxia, C‐Caspas: cleaved‐caspase. * VS Con, # VS BPD. The concentration of HBM‐Exo: 15.4 μg/ml protein

The activation of the IL‐17 signalling pathway by the addition of IL‐17A cytokine resulted in the disappearance of the effects of HBM‐Exo on proliferation inhibition, apoptosis increase and SPC down‐regulation in AT‐II cells induced by high oxygen exposure. (Figure 8A‐C). These results suggest that HBM‐Exo might inhibit IL‐17 signalling pathway, inhibit cell apoptosis and reducing AT‐II cell damage, thus playing a role in the prevention and treatment of BPD.

## 4 DISCUSSION

BPD has a high morbidity and mortality rate in NICU and may develop into long‐term consequences in adulthood.^25^ However, the molecular pathways and cellular mechanisms that affect the pathophysiology and progression of BPD are limited. Some studies showed that an important cause of BPD is oxidative stress,^26^ which may be caused by several mechanical ventilation and infection.^27^, ^28^ Early cumulative oxygen exposure is independently associated with BPD.^29^

A previous study indicated that HBM feeding can reduce the incidence of BPD.^14^, ^15^, ^30^ HBM has antioxidant components, to reduce oxidative stress in premature infants and reduce the occurrence of BPD.^31^ The HBM‐Exo can contain large amounts of antioxidants that are protected from digestion and absorption by the gastrointestinal tract, thus entering the epithelial cells of the lung to against lung damage. Colin et al. reported that HBM‐Exo protect cells from oxidative stress‐induced cell death.^16^ Till date, few studies have been conducted on the function of HBM‐Exo in patients with BPD and their potential role in the development of BPD. In this study, we found HBM‐Exo reduce AT II cell damage and improve lung injury caused by hyperoxia exposure in newborn SD rats,

Il‐17A is produced by a wide range of innate immune cells and has different pro‐inflammatory effects.^32^ γδT cells in mice produce IL‐17 in the embryonic thymus only on day 15.^33^ This period is equivalent to 24–28 weeks of human gestation, during which lung development is not mature, and the increase of IL‐17A activates the IL‐17 signalling pathway, causing inflammation, which may further affect lung development, which lead to the occurrence of BPD.^34^ Recent studies found that the expression of IL‐17A is significantly up‐regulated in the external circulation of children with BPD, and the IL‐17 signalling pathway is significantly activated.^35^, ^36^ Therefore, our hypothesis was that the activation of IL‐17 signalling pathway may play an important regulatory role in the occurrence and development of BPD. Several experimental studies showed that the activation of the IL‐17 signalling pathway promotes apoptosis.

The prevention and cure of BPD through the IL‐17 signalling pathway becomes a new problem to think about in the future. Many previous studies confirmed that the activation of IL‐17 signalling pathway promotes cell apoptosis.^37^, ^38^. Apoptosis of alveolar epithelial cells plays an important role in the occurrence and development of BPD.^39^, ^40^ Therefore, our speculation was that HBM‐Exo might prevent and cure BPD by inhibiting the IL‐17 signalling pathway to reduce the apoptosis of alveolar epithelial cells. The FADD gene is a key target gene downstream of the IL‐17 signalling pathway. A study showed that the activation of IL‐17 signalling pathway can promote the expression of its downstream target gene FADD and activate caspase‐mediated apoptosis.^41^ The caspase protein family plays a particularly important role in the occurrence and development of cell apoptosis. Caspase 3 is the main executor of apoptosis,^42^ and caspase 9 is the upstream regulator of caspase 3 and can initiate caspase cascade to regulate apoptosis.^43^ Therefore, our speculation was that HBM‐Exo could inhibit the expression of FADD by inhibiting the IL‐17 signalling pathway, thus inhibiting the expression of activated apoptotic proteins, so as to repair the injury of AT‐II cells. This study revealed that FADD was consistently highly expressed in BPD clinical lung tissue samples, animal models and AT II cell models that exposed to hyperoxia. HBM‐Exo treatment reduced the expression of FADD downstream of the IL‐17 signalling pathway, as well as that of C‐caspase 3 and C‐caspase 9 induced by high oxygen exposure. In addition, salvage experiments showed that the inhibition of HBM‐Exo on FADD and C‐Caspase 3 and C‐Caspase 9 was also decreased after the activation of the IL‐17 signalling pathway. Phenotypic analysis showed that HBM‐Exo had no repair effect on AT‐II cell viability, apoptosis and SPC injury phenotype induced by high oxygen exposure after activation of IL‐17 signalling pathway. The above experimental evidence confirmed that HBM‐Exo might inhibit the expression of the downstream target gene FADD by inhibiting the IL‐17 signalling pathway, thereby reducing cell apoptosis, finally repairing the damage of alveolar epithelial cells, thus playing a role in the prevention and treatment of BPD.

HBM‐Exo carries proteins, lipids, RNA and other active components, and can transport these components to target organs for use.^44^ HBM‐Exo and its small RNA, other active ingredients were not detected in formula milk^45^; the active ingredients carried by HBM‐Exo were different from those in HBM. Proteomics found that 633 protein molecules in HBM‐Exo did not exist in HBM suggesting that HBM‐Exo could play a unique function.^46^ Therefore, the research group will further analyse the composition of HBM‐Exo in the later stage, and further explore what kind of substances transported by HBM‐Exo play a protective role in BPD.

There are some limitations in our research. Ideally, we would like to confirm the RNA‐Seq data obtained in MLE‐12 cells rather than hominid alveolar type II cells. However, these cells are difficult to obtain, and the cells should be freshly isolated from human lung tissue. In addition, although our results showed that HBM‐Exo inhibited cell apoptosis and promoted their proliferation through the IL‐17 signalling pathway, other pathways may also be involved in this regulation. The concentration of EGF is higher in human colostrum.^47^, ^48^ Importantly, Holder pasteurization, which is commonly used for the treatment of donor breast milk, does not reduce EGF concentration. EGF has also been found to play an important role in neonatal disease.^49^, ^50^ VEGF, a growth factor, is present in human milk at a much higher concentration than in human serum,^51^ and a growing body of literature suggests that it may contribute to pulmonary hypertension, which is usually associated with bronchopulmonary dysplasia. Our KEGG Pathway analysis also showed that VEGF signalling pathway was significant involved, and our research group will continue to conduct in‐depth exploration on VEGF in the later stage.

## 5 CONCLUSION

In conclusion, our study is the first reporting the role of HBM‐Exo on BPD. HBM‐Exo promoted the proliferation of AT II cells and inhibited their apoptosis by regulating the IL‐17 signalling pathway, thus exerting a protective effect on BPD. Since exosomes are a new type of in‐milk regulator with good application value, our results may help in the preventing of BPD, providing better suggestions for the nutrition of newborns.

## AUTHOR CONTRIBUTION

**Yahui Zhou:** Validation (equal); Writing – original draft (equal). **Yiwen Liu:** Writing – original draft (supporting). **Gen Xu:** Validation (supporting). **Lingjie Liu:** Data curation (supporting). **Huimin Li:** Data curation (supporting); Software (supporting). **Yubai Li:** Validation (equal). **Jing Yin:** Funding acquisition (equal); Resources (equal). **Xingyun Wang:** Funding acquisition (equal); Methodology (equal); Writing – review & editing (equal). **Zhangbin Yu:** Project administration (equal); Writing – review & editing (equal).

## CONFLICT OF INTEREST

No conflict of interest.

## Supporting information

Fig S1

Table S1

## Data Availability

The data that support the findings of this study are available from the corresponding author upon reasonable request.
