# Supplementary figures and images for: Human breast milk‐derived exosomes through inhibiting AT II cell apoptosis to prevent bronchopulmonary dysplasia in rat lung
Source: J Cell Mol Med. 2022 Jul 14;26(15):4169–82. doi: 10.1111/jcmm.17334 (PMC9344832; doi:10.1111/jcmm.17334)

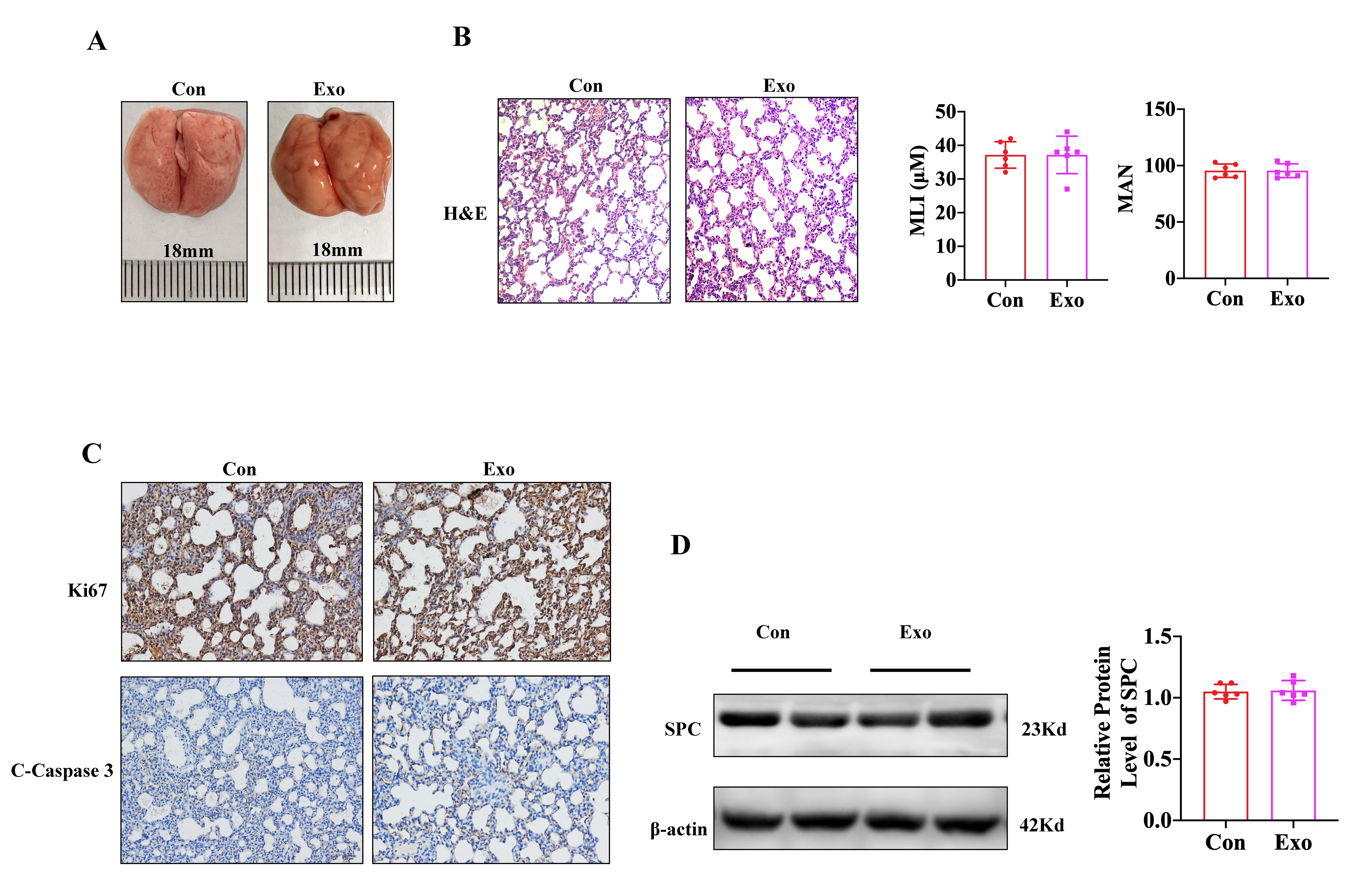

Supplement: Supplementary file 1 — Fig S1 [file JCMM-26-4169-s001.tif]
