# Supplementary material for: Human breast milk‐derived exosomes through inhibiting AT II cell apoptosis to prevent bronchopulmonary dysplasia in rat lung
Source: J Cell Mol Med. 2022 Jul 14;26(15):4169–82. doi: 10.1111/jcmm.17334 (PMC9344832; doi:10.1111/jcmm.17334)
Supplement: Supplementary file 2 — Table S1 [file JCMM-26-4169-s002.docx]

**Supplementary table 1 The different genes were expressed in the hyperoxia group and hyperoxia with HBM-Exo group**

| **Gene iId** | **Gene Name** | **Description** | **Log2FC** | **P value** |
| --- | --- | --- | --- | --- |
| **Up** |  |  |  |  |
| 107977570 | LOC107977570 | PDZ and LIM domain protein 5-like | 7.48640839 | 3.27E-35 |
| 100750538 | Bin2 | bridging integrator 2 | 4.63910399 | 2.47E-06 |
| 100771465 | Npnt | nephronectin | 3.89524449 | 0.00236325 |
| 100751872 | Plcd4 | phospholipase C delta 4 | 2.88351822 | 7.90E-05 |
| 100765712 | Eaf2 | ELL associated factor 2 | 2.76443897 | 1.02E-06 |
| 100760214 | Mfsd3 | major facilitator superfamily domain containing 3 | 2.61755119 | 3.00E-05 |
| 103164015 | LOC103164015 | uncharacterized LOC103164015 | 2.58727027 | 0.0001446 |
| 100773170 | Hao2 | hydroxyacid oxidase 2 | 2.55779336 | 0.00217613 |
| 100750531 | Vwf | von Willebrand factor | 2.49622103 | 0.00046029 |
| 100755852 | Egr1 | early growth response 1 | 2.38785031 | 1.39E-08 |
| 113831595 | LOC113831595 | - | 2.35661672 | 0.00017591 |
| 107977041 | LOC107977041 | zinc finger protein 728-like | 2.19028183 | 0.00038674 |
| 100753037 | Cep290 | centrosomal protein 290 | 1.90679081 | 0.00162144 |
| 100758084 | Liph | lipase H | 1.80772578 | 0.00067404 |
| 100753619 | Ccdc28b | coiled-coil domain containing 28B | 1.75134463 | 0.00071488 |
| 103161373 | LOC103161373 | uncharacterized LOC103161373 | 1.71675307 | 0.00010418 |
| 100755669 | Foxs1 | forkhead box S1 | 1.6956851 | 2.80E-06 |
| 100759809 | Nlrp3 | NLR family pyrin domain containing 3 | 1.68346792 | 0.00245602 |
| 100752735 | Col12a1 | collagen type XII alpha 1 chain | 1.63041714 | 3.24E-06 |
| 100758741 | Plagl1 | PLAG1 like zinc finger 1 | 1.56887293 | 8.36E-07 |
| 100753477 | Myl9 | myosin light chain 9 | 1.52994753 | 1.13E-07 |
| 103161319 | Znf383 | zinc finger protein 383 | 1.52128007 | 0.00186576 |
| **Down** |  |  |  |  |
| 100755382 | Fhit | fragile histidine triad | -3.1319674 | 6.77E-05 |
| 113835261 | LOC113835261 | - | -2.7908847 | 9.18E-05 |
| 100770411 | Tmem220 | transmembrane protein 220 | -2.4410756 | 0.00162507 |
| 103163338 | LOC103163338 | uncharacterized LOC103163338 | -2.3221473 | 2.27E-11 |
| 100751745 | Atoh8 | atonal bHLH transcription factor 8 | -2.246128 | 2.58E-07 |
| 100752022 | Thbs2 | thrombospondin 2 | -1.9413776 | 0.00022442 |
| 100768837 | Rab5b | RAB5B, member RAS oncogene family | -1.8386209 | 1.46E-06 |
| 100751577 | Id3 | inhibitor of DNA binding 3, HLH protein | -1.8010873 | 7.42E-62 |
| 113832035 | LOC113832035 | - | -1.7700103 | 0.00116169 |
| 103164338 | LOC103164338 | uncharacterized LOC103164338 | -1.6833576 | 1.44E-10 |
| 100769963 | Scn4a | sodium voltage-gated channel alpha subunit 4 | -1.5536653 | 1.87E-08 |
| 100767726 | Cd24 | CD24 molecule | -1.5431291 | 0.00215427 |
